# Supplementary material for: Deletion of wfs1 Impairs Oligodendrocyte Precursor Cells Dorsal Distribution and Myelination Through the wfs1-hmgcs1 Axis in Zebrafish
Source: Biology (Basel). 2026 Aug 16;15(16):1402. doi: 10.3390/biology15161402 (PMC13510151; doi:10.3390/biology15161402)
Supplement: Supplementary file 1 [file biology-15-01402-s001.zip › File S1-Transcriptome sequencing data and differentially expressed genes (DEGs) in wfs1-- zebrafish at 5 dpf/1.GO/wfs1KOvsWT/GSEA/WT/EXTRACELLULAR_MATRIX_ASSEMBLY/EXTRACELLULAR_MATRIX_ASSEMBLY(GO_0085029).html]

Details for gene set EXTRACELLULAR\_MATRIX\_ASSEMBLY(GO:0085029)[GSEA]


|  || Dataset | deg.sample |
| Phenotype | sample.cls |
| Upregulated in class | WT |
| GeneSet | EXTRACELLULAR\_MATRIX\_ASSEMBLY(GO:0085029) |
| Enrichment Score (ES) | -0.4977328 |
| Normalized Enrichment Score (NES) | -1.7043563 |
| Nominal p-value | 0.0 |
| FDR q-value | 0.3282643 |
| FWER p-Value | 0.89 |
Table: GSEA Results Summary

  

Fig 1: Enrichment plot: EXTRACELLULAR\_MATRIX\_ASSEMBLY(GO:0085029)
  
Profile of the Running ES Score & Positions of GeneSet Members on the Rank Ordered List

  

| PROBE | DESCRIPTION   (from dataset) | GENE SYMBOL | GENE\_TITLE | RANK IN GENE LIST | RANK METRIC SCORE | RUNNING ES | CORE ENRICHMENT || 1 | 100126131 | zgc:171687 |  |  | 1322 | 1.791 | 0.0224 | No |
| 2 | 405825 | mfap4 |  |  | 1736 | 1.515 | 0.0635 | No |
| 3 | 100538113 | si:ch1073-110a20.1 |  |  | 3288 | 0.976 | 0.0496 | No |
| 4 | 100004613 | tmeff2a |  |  | 3983 | 0.834 | 0.0576 | No |
| 5 | 449806 | fbln5 |  |  | 5564 | 0.631 | 0.0305 | No |
| 6 | 260350 | has2 |  |  | 5757 | 0.611 | 0.0463 | No |
| 7 | 282555 | has3 |  |  | 5960 | 0.591 | 0.0611 | No |
| 8 | 100538070 | si:ch1073-110a20.2 |  |  | 6265 | 0.561 | 0.0715 | No |
| 9 | 403130 | has1 |  |  | 6975 | 0.494 | 0.0669 | No |
| 10 | 286831 | lamb4 |  |  | 10456 | 0.275 | -0.0325 | No |
| 11 | 337767 | lamb2 |  |  | 11883 | 0.197 | -0.0703 | No |
| 12 | 561440 | ntn4 |  |  | 12611 | 0.159 | -0.0874 | No |
| 13 | 286830 | lamb1a |  |  | 12912 | 0.145 | -0.0917 | No |
| 14 | 566851 | tmem2 |  |  | 14278 | 0.079 | -0.1317 | No |
| 15 | 100537823 | si:ch1073-110a20.3 |  |  | 14420 | 0.073 | -0.1335 | No |
| 16 | 118437 | gpc4 |  |  | 14777 | 0.057 | -0.1426 | No |
| 17 | 386787 | itga5 |  |  | 14892 | 0.051 | -0.1444 | No |
| 18 | 560014 | ntng2a |  |  | 15354 | 0.030 | -0.1578 | No |
| 19 | 494044 | efemp2a |  |  | 15451 | 0.025 | -0.1599 | No |
| 20 | 386710 | mibp2 |  |  | 15802 | 0.006 | -0.1707 | No |
| 21 | 566796 | lama4 |  |  | 18438 | -0.096 | -0.2500 | No |
| 22 | 569075 | ntng1a |  |  | 20302 | -0.179 | -0.3021 | No |
| 23 | 373882 | mmp14a |  |  | 22032 | -0.269 | -0.3468 | No |
| 24 | 108180329 | thbd |  |  | 22955 | -0.324 | -0.3642 | No |
| 25 | 556605 | lamb1b |  |  | 23520 | -0.362 | -0.3689 | No |
| 26 | 797681 | tmeff2b |  |  | 24763 | -0.436 | -0.3924 | No |
| 27 | 321298 | col14a1a |  |  | 25940 | -0.524 | -0.4106 | No |
| 28 | 553337 | lamb2l |  |  | 26254 | -0.552 | -0.4007 | No |
| 29 | 100329793 | si:dkey-234i14.3 |  |  | 29346 | -1.013 | -0.4616 | Yes |
| 30 | 101885039 | ntng2b |  |  | 29352 | -1.014 | -0.4256 | Yes |
| 31 | 566945 | mmp14b |  |  | 30862 | -1.822 | -0.4079 | Yes |
| 32 | 100007462 | si:zfos-2330d3.1 |  |  | 31218 | -2.257 | -0.3385 | Yes |
| 33 | 335430 | wu:fj11g02 |  |  | 31501 | -2.927 | -0.2429 | Yes |
| 34 | 100126122 | zgc:173915 |  |  | 31599 | -3.255 | -0.1298 | Yes |
| 35 | 393502 | zgc:66455 |  |  | 31729 | -3.891 | 0.0051 | Yes |
Table: GSEA details
[plain text format]

  

Fig 2: EXTRACELLULAR\_MATRIX\_ASSEMBLY(GO:0085029)
  
Blue-Pink O' Gram in the Space of the Analyzed GeneSet

  

Fig 3: EXTRACELLULAR\_MATRIX\_ASSEMBLY(GO:0085029): Random ES distribution
  
Gene set null distribution of ES for
 **EXTRACELLULAR\_MATRIX\_ASSEMBLY(GO:0085029)**
